# Whole genome demographic models indicate divergent effective population size histories shape contemporary genetic diversity gradients in a montane bumble bee

**DOI:** 10.1002/ece3.9778

**Published:** 2023-01-31

**Authors:** Jeffrey D. Lozier, James P. Strange, Sam D. Heraghty

**Affiliations:** ^1^ Department of Biological Sciences The University of Alabama Tuscaloosa Alabama USA; ^2^ Department of Entomology The Ohio State University Columbus Ohio USA

**Keywords:** bumble bees, effective population size, elevation, latitude, MSMC2, PSMC, sequentially Markovian coalescent, species distribution models

## Abstract

Understanding historical range shifts and population size variation provides an important context for interpreting contemporary genetic diversity. Methods to predict changes in species distributions and model changes in effective population size (*N*_e_) using whole genomes make it feasible to examine how temporal dynamics influence diversity across populations. We investigate *N*_*e*_ variation and climate‐associated range shifts to examine the origins of a previously observed latitudinal heterozygosity gradient in the bumble bee *Bombus vancouverensis* Cresson (Hymenoptera: Apidae: *Bombus* Latreille) in western North America. We analyze whole genomes from a latitude‐elevation cline using sequentially Markovian coalescent models of *N*_e_ through time to test whether relatively low diversity in southern high‐elevation populations is a result of long‐term differences in *N*_e_. We use Maxent models of the species range over the last 130,000 years to evaluate range shifts and stability. *N*_e_ fluctuates with climate across populations, but more genetically diverse northern populations have maintained greater *N*_e_ over the late Pleistocene and experienced larger expansions with climatically favorable time periods. Northern populations also experienced larger bottlenecks during the last glacial period, which matched the loss of range area near these sites; however, bottlenecks were not sufficient to erode diversity maintained during periods of large *N*_e_. A genome sampled from an island population indicated a severe postglacial bottleneck, indicating that large recent postglacial declines are detectable if they have occurred. Genetic diversity was not related to niche stability or glacial‐period bottleneck size. Instead, spatial expansions and increased connectivity during favorable climates likely maintain diversity in the north while restriction to high elevations maintains relatively low diversity despite greater stability in southern regions. Results suggest genetic diversity gradients reflect long‐term differences in *N*_e_ dynamics and also emphasize the unique effects of isolation on insular habitats for bumble bees. Patterns are discussed in the context of conservation under climate change.

## Graphical abstract

We use whole genome demographic modeling to demonstrate that existing patterns of genetic diversity across latitudes in a montane bumble bee are shaped by historical differences in population size dynamics. High‐diversity populations have a history of larger effective population sizes through time, even with larger losses in habitat suitability during glacial periods resulting in larger Pleistocene bottlenecks. Results suggest that southern high‐elevation restricted populations have a reduced maximum population size through time than northern low‐elevation populations despite greater niche stability, which has implications for conserving genetic diversity in these more isolated regions under climate change.

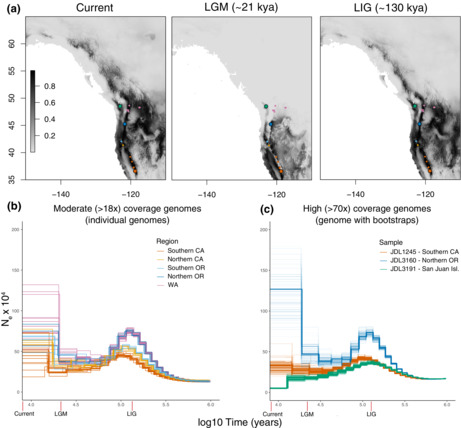

## 1 INTRODUCTION

Populations in complex landscapes exhibit differences in diversity and connectivity driven by the distribution of habitable areas (Holderegger & Wagner, 2008; McRae & Beier, 2007; Storfer et al., 2007). Changes in genetic diversity from human‐associated habitat alteration are an increasing concern, and factors like landscape modification and climate change have been associated with reduced genetic diversity and connectivity (Epps et al., 2006; Jha, 2015; Rubidge et al., 2012; Struebig et al., 2011). However, genetic variation is also influenced by the legacy of historical events that have driven fluctuations in effective population size (*N*_e_). The impact of Quaternary climate variation on genetic diversity is well‐recognized (Hewitt, 2000), and recent studies have more explicitly examined links between species range stability and genetic diversity (Carnaval et al., 2009; Garrick et al., 2021; Koch et al., 2018). Revealing how landscape changes drive declines and recovery in *N*_e_ has thus become important for reconstructing evolutionary histories, but also for interpreting contemporary genetic diversity and making predictions about vulnerability to environmental change.

Genomes contain substantial information about historical demography. The development of methods to reconstruct historical demography using whole genomes even from single individuals (e.g., Li & Durbin, 2011; Schiffels & Durbin, 2014) has thus facilitated high‐resolution temporal analyses in wild populations (Mather et al., 2020; Nadachowska‐Brzyska et al., 2016; Spence et al., 2018). Sequentially Markovian coalescent (SMC) methods (Mather et al., 2020), in particular, have gained favor for rapidly modeling *N*_e_ trajectories because these methods can reveal demographic history for a population using only a single diploid genome sequence, although can also be applied with population sampling (Foote et al., 2021; Nadachowska‐Brzyska et al., 2016; Taylor et al., 2021). Essentially, SMC models estimate the time to the most recent common ancestor for local genealogies at each locus in the genome as defined by ancestral recombination events (Li & Durbin, 2011; Schiffels & Wang, 2020). By providing estimates of *N*_e_ changes over time, as opposed to providing a single‐point estimate of diversity (e.g., current heterozygosity), SMC models are a powerful tool for examining how climate fluctuations may influence genetic variation (Taylor et al., 2021).

Bumble bees (*Bombus*) are abundant insects in terrestrial biomes, where they provide important pollination services (Goulson, 2010; Williams, 1998). Many bumble bee species have experienced declines (Cameron & Sadd, 2020). Studies have investigated how genetic diversity varies across species with different histories of decline and in different landscapes (Cameron et al., 2011; Ellis et al., 2006; Goulson et al., 2011; Koch et al., 2018; Lozier, 2014; Lozier et al., 2011; Martinet et al., 2018), as well as how climate influences decline risk (Iserbyt & Rasmont, 2012; Kerr et al., 2015; Martinet et al., 2020; Rasmont et al., 2015). In North America, bumble bees have high species richness in mountainous regions (e.g., Koch et al., 2015), facilitated by numerous thermal adaptations (Heinrich, 2004). The evolution of montane bumble bees has received increasing attention (Christmas et al., 2021; Jackson et al., 2018, 2020; Lee et al., 2019; Montero‐Mendieta et al., 2019; Williams et al., 2015, 2018), driven by data demonstrating threats from climate change for these species (Biella et al., 2017; Marshall et al., 2020; Pradervand et al., 2014). Thus, examining how populations historically responded to climate fluctuations would be useful for better‐understanding threats from environmental change even for species that remain common (Dellicour et al., 2017; Koch et al., 2018).

**FIGURE 1:**
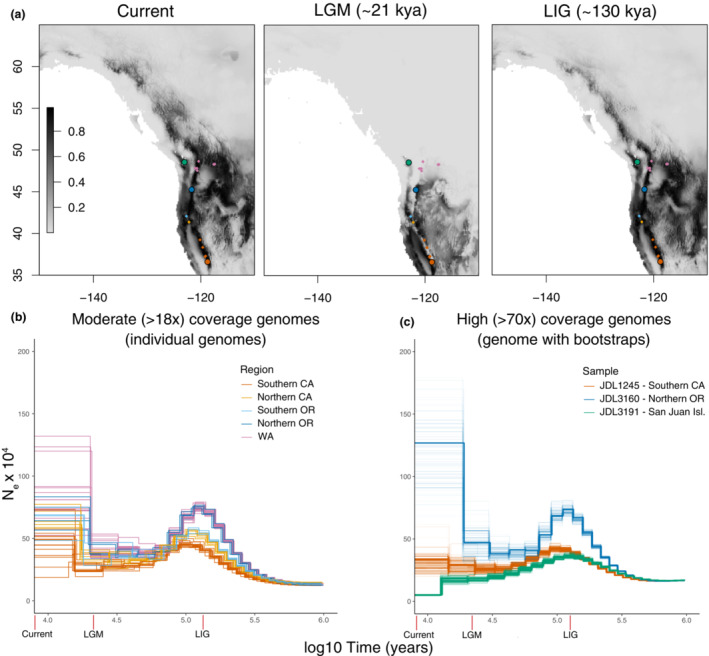
(a) Maxent model maps for current climate, last glacial maximum (LGM) climate, and last interglacial (LIG) climate for *B. vancouverensis* in western North America. Shading indicates Maxent clog‐log suitability values from low (light) to high (dark) suitability. Sampling sites for moderate coverage genomes are shown as small points, while larger points reflect locations for three high coverage genomes. Points are color coded by focal region and are used throughout the manuscript. (b and c) PSMC plots of effective population size (*N*_e_) over time for (b) 42 individual moderate coverage (>18x, mean of 22.5x) genomes and (c) three high coverage (77‐82x coverage) color coded by region.

*Bombus vancouverensis* is common in western North America (Ghisbain et al., 2020; Stephen, 1957) and has been a focus of several population genetic studies (Christmas et al., 2021; Jackson et al., 2018, 2020; Lozier et al., 2013, 2016, 2021). The population genetic structure of *B. vancouverensis* is well understood from prior analyses of mitochondrial sequences, microsatellites, and RAD‐tag sequences (Ghisbain et al., 2020; Jackson et al., 2018; Lozier et al., 2011, 2013). This study focuses on a latitudinal transect across westernmost *B. vancouverensis* populations (California, Oregon, Washington, USA; Figure 1), which provides an elevational gradient from zero to >2900 m above sea level and represents a morphologically and partly genetically distinct lineage (Ghisbain et al., 2020). This region is interesting because these populations are weakly structured (global *F*_ST_ = ~0.02) but exhibit a clear latitude‐altitude gradient in genetic diversity, with diversity lower in southern high‐elevation populations while northern populations appear more well‐connected with higher heterozygosity (Jackson et al., 2018). Another interesting aspect of *B. vancouverensis* in the region is the presence of populations on islands in the Pacific Northwest. Islands are well known to accelerate genetic signatures of isolation in bumble bees (Goulson et al., 2011; Jha, 2015; Lozier et al., 2011) and can serve as a reference to test whether SMC models can detect effects of recent discrete fragmentation on genome variation.

In this study, we take advantage of existing whole genome data from prior studies (Ghisbain et al., 2020; Heraghty et al., 2020, 2022) to address new hypotheses relating to the origins of latitudinal genetic diversity gradients in *B. vancouverensis*. Grounded in prior knowledge about latitudinal patterns of genetic diversity and structure across the region, we combine demographic models from representative whole genome sequences with temporal species distribution models to examine how the history of climatic fluctuations has shaped range dynamics and genetic diversity. We refine estimates of spatial variation in heterozygosity using the resolution afforded by whole genome data and then employ SMC modeling to estimate changes in *N*_e_ over time using individual and population‐level genome sequences. We also include a genome from an insular population to evaluate signatures of recent isolation in SMC models. We use extensive occurrence data to model the *B. vancouverensis* range over the last ~130,000 years and examine how *N*_e_ changes are associated with fluctuations in range size and stability. We investigate several related hypotheses. (1) If current genetic diversity differences observed among populations can be explained by long‐term Quaternary *N*_e_ differences, *N*_e_ in less diverse southern populations will be persistently smaller than in northern populations through time. (2) If more recent *N*_e_ declines can be detected with SMC models, insular genome data should demonstrate strong contemporary bottlenecks. (3) If bumble bee populations can rapidly respond to expansions and contractions of the geographic range, temporal fluctuations in population size in individual genomes should be mirrored by variation in the spatial extent of the species' niche.

## 2 METHODS

### 2.1 Whole genome data

Whole genomes for *B. vancouverensis* were obtained from previously collected samples that were used for investigations of taxonomy, assembly of reference genomes, or analyses of environment‐genome associations (Ghisbain et al., 2020; Heraghty et al., 2020, 2022) (Figure 1, see Appendix S1, Tables S1 and S2). We combine these previously published whole genome data to conduct novel statistical analyses relating to the history of previously reported latitude‐altitude genetic diversity clines in this species (Jackson et al., 2018; Lozier et al., 2011). All bees included here are female diploids and should represent distinct colonies (Jackson et al., 2018). Briefly, most genomes consist of 150 bp paired‐end Illumina libraries sequenced to approximately 20‐25x on the NovaSeq 6000 S4 platform (Illumina) (Heraghty et al., 2022). We also incorporated available data from three genomes that were sequenced to higher coverage (76.7x–81.9x; Tables S1 and S2) (see below). Sequences were filtered using default parameters in bbduk (Bushnell, 2020; http://sourceforge.net/projects/bbmap/) and mapped to the *B. vancouverensis* genome (NCBI RefSeq GCF_011952275.1) with bwa‐mem v0.7.15‐r1140 (Li, 2013). picard tools v2.20.4 (Broad Institute, 2019; http://broadinstitute.github.io/picard/) was used to remove PCR duplicates and sort and index BAM files. We created a BED file to only retain positions on large scaffolds (>500 kb, *n* = 89), which can be beneficial for SMC methods (Schiffels & Wang, 2020) (234.87 Mb of sequence retained, representing most of the expected bumble bee genome size of ~250–280 Mb). Some analyses require a “mappability mask” including regions uniquely mappable by short reads, which we created with snpable regions (lh3lh3.users.sourceforge.net/snpable.shtml) followed by ‘makeMappabilityMask.py’ from msmc tools (https://github.com/stschiff/msmc‐tools/blob/master/README.md) (Schiffels & Durbin, 2014; Schiffels & Wang, 2020).

**FIGURE 2:**
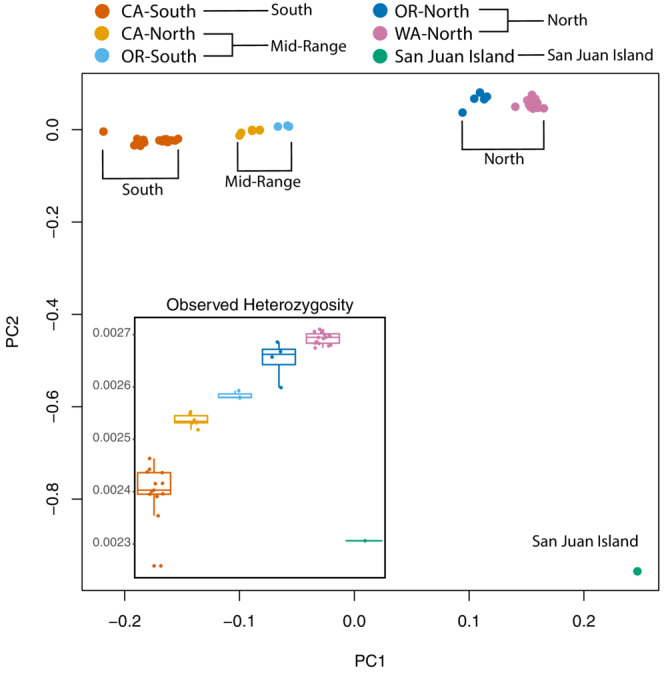
pcangsd principal component analysis for sequenced *B. vancouverensis* genomes from all focal regions (Figure 1a) with (inset) boxplots of regional diversity (individual heterozygosity bp^−1^ bee^−1^) from the site frequency spectrum estimated by ANGSD.

Based on recommendations for SMC methods we only used samples with a mean coverage >18x (Nadachowska‐Brzyska et al., 2016) estimated using samtools v1.10 (Li et al., 2009). Sequencing depth was consistent across samples (*N* = 42 moderate coverage genomes, mean ± SD coverage = 22.5x ± 3.4x; Table S2). Based on initial results using individuals (see Results), for some analyses mainland samples were grouped into three regional populations (Figures 1 and 2, Tables S1 and S2; southern = southern California, mid‐range = northern California + southern Oregon, northern = northern Oregon + Washington).

### 2.2 Basic diversity statistics and site frequency Spectrum estimation

angsd v0.935‐53‐gf475f10 (Korneliussen et al., 2014) was used to estimate population genetic statistics accounting for genotype uncertainty (Korneliussen et al., 2013; Nielsen et al., 2012). We estimated the folded site frequency spectrum (SFS) (Nielsen et al., 2012) across >500 kb scaffolds for the three population regions using the ‐doSfs command, with minimum mapping and base quality of 20, mapping quality downgrading of C = 50, gatk (McKenna et al., 2010) genotype likelihoods GL = 2, and base quality recalibration baq = 1, followed by realsfs (specifying ‐fold 1). We used the SFS for all samples in each population group for diversity calculations, but for plotting single population SFS, we also performed calculations for nine randomly selected individuals per region so that the SFS would have the same bin sizes. We determined the two estimates of θ (assuming θ = 3*N*_e_ *μ* for haplodiploid bees, which have 1.5 allele copies per individual; Goulson et al., 2008) based on pairwise differences and segregating sites (θ_π_ and θ_W_, respectively) and Tajima's *D* for each scaffold (Korneliussen et al., 2013). Values were scaled by the number of sequenced bases to obtain per site values. We compared diversity among southern, mid‐range, and northern regions using linear mixed models conducted with r v4.0.5 (R Core Team, 2021) package ‘lme4’ 1.1–27 (Bates et al., 2015) with the region as a fixed effect and scaffold specified as a random effect. *P*‐values were determined with ‘lmerTest’ 3.1–3 using the Swatterwhaithe method (Kuznetsova et al., 2017), and a likelihood ratio test was used to compare the region model against a random effect‐only model. Individual heterozygosites were determined using a folded SFS (‐doSFS and realsfs commands, as above) from each sample (the “1” count bin in the SFS divided by total counts). For each region pair, we also estimated the folded 2D SFS and weighted *F*_ST_ (not performed for San Juan Island genome with only one sample). Visualization of population structure was performed using PCA with pcangsd v1.03 (Meisner & Albrechtsen, 2018).

### 2.3 Markovian coalescent demographic analyses

#### 2.3.1 PSMC

The Pairwise Sequentially Markovian Coalescent (PSMC) model (Li & Durbin, 2011) is a widely used (Foote et al., 2021; Mather et al., 2020; Taylor et al., 2021) SMC methods that estimate the distribution of coalescent times between two unphased chromosomes from a single genome to infer *N*_e_ over a series of intervals back in time. PSMC inputs were generated using samtools mpileup, with minimum mapping quality set to q = 20, base quality set to Q = 20, the mapping quality downgrading parameter set to C = 50, and the 500 kb scaffold bed file to specify target regions. Conversion to the consensus diploid genome was performed using bcftools call, followed by conversion to fastq using the samtools ‘vcfutils.pl vcf2fq’ script, setting sites with minimum root‐mean‐squared mapping quality (Q) < 20, within 10 bp of an insertion–deletion, and below or above coverage thresholds (d or D, respectively) as missing data. Samples were sequenced to similar coverage so we specified a minimum depth of d = 10 and a maximum depth of D = 50 (see below for details on high coverage samples that required alteration of D).

Default settings for the atomic time interval parameter −p = “4 + 25*2 + 4 + 6” were designed for human analyses (Li & Durbin, 2011) but are expected to work well for many organisms (Patil & Vijay, 2021). We modified other parameters to ensure at least 20 recombination events per interval. We set initial ‐t (maximum TMRCA) at 7 and initial ‐r (ratio of scaled mutation to recombination rate θ/ρ) at 2, although the shape of outputs varied little with changes to these parameters, as expected (Mather et al., 2020). For plotting, we used a generation time of one generation per year and a direct mutation rate estimate of 3.6 × 10^−9^ per site per year (Liu et al., 2017). Values converted to “real world” time and *N*_e_ were extracted using the psmc_plot.pl script (modified to rescale using 3*N*_e_ *u*) with the ‐R option and plotted using geom_step of ‘ggplot2’ 3.3.5 (Wickham, 2016) in r.

We also had the three higher coverage genomes (Tables S1 and S2) available that represented the northern (JDL3160, 76.7x depth) and southern (JDL1245, 81.9x depth; Heraghty et al., 2020) regions, as well as the sample collected from the San Juan Island population for which we had previously identified unusual genetic variation with microsatellites (Lozier et al., 2011) (JDL3191, 79.7x depth; Ghisbain et al., 2020). We used these samples to examine coverage effects on PSMC estimates and to confirm results from lower coverage samples. The San Juan Island genome was also used to test the hypothesis that recent population size changes would be detectable with SMC methods. For psmc, we specified d = 10 and D = 150 for generating the consensus fastq with the samtools mpileup, bcftools call, and ‘vcfutils.pl vcf2fq’ pipeline as described above. We also performed bootstrapping to examine variance in PSMC estimates for each atomic interval. For bootstraps, we divided scaffolds into 100 kb units and performed psmc analyses on 100 resampled data sets using the provided example script.

#### 2.3.2 MSMC2

Although we primarily present results from psmc, ensuring that results are comparable across methods can strengthen inferences (Mather et al., 2020). msmc2 is similar to psmc but can use population‐level data (Schiffels & Wang, 2020). We focus on comparisons of *N*_e_ for the mainland regions, as San Juan Island did not have population‐level sequencing. Given similar individual trajectories for spatially proximate genomes in PSMC, genomes from some nearby sites were combined for MSMC2 to increase population samples (6–10 haplotypes each). Data files were prepared with minor modifications of scripts from J Rick (https://github.com/jessicarick/msmc2_scripts). MSMC2 also required the mappability mask. Separate VCF files for each scaffold and individual were created using samtools and bcftools as above and excluding sites with <10x coverage. Mask BED files representing sequenced sites for each sample and scaffold required for MSMC2 were created using ‘bamcaller.py’ in the MSMC tools repository. Input files for populations were prepared using VCFs, individual masks, and the masked reference genome using “generate_multihetsep.py” in MSMC tools. MSMC2 was run setting the ‐I parameter to only evaluate coalescence between haplotypes within individuals for each population (Schiffels & Wang, 2020) and using the default time interval parameter (−p = “1*2 + 25*1 + 1*2 + 1*3”); however, initial results suggested that the default ‐p time interval setting produced massive variance among populations and several unrealistically large *N*_e_ estimates at the most recent time interval (<2500 years before present, or ybp). Such spikes are a typical sign of model overfitting (Schiffels & Wang, 2020) and we used a less complex setting of −p = “1*2 + 16*1 + 1*2” recommended by Schiffels and Wang (2020) to eliminate the *N*_e_ spikes without notably altering curve shapes within the main period of interest. We converted outputs to real world time and *N*_e_ for plotting as above.

### 2.4 Distribution modeling

To predict changes in habitable areas during the Pleistocene and Holocene we used species distribution models from the presence‐only “maximum entropy” method maxent (Elith et al., 2011; Phillips et al., 2006). We obtained occurrence data for *B. vancouverensis* from the Geographic Biodiversity Informatics Facility (GBIF.org, search date November 14, 2021), using records from the USDA‐ARS Pollinating Insect‐Biology, Management, Systematics Research Collection, the National Museum of Natural History Collection, the Smithsonian Institution Collection, the Canadian Biodiversity Information Facility Collection, the Illinois Natural History Survey Collection, and the Yale University Peabody Museum Collection (Canadian Biodiversity Information Facility, 2021; Dmitriev, 2015; Gall, 2021; Ikerd, 2019; Orrell, 2021; Strange, 2019; Strange et al., 2021). We downloaded records with geographic coordinates for *Bombus bifarius* Cresson that span the contiguous USA, Canada, and Alaska, as *B. vancouverensis* was only recently recognized as a distinct species (Ghisbain et al., 2020). We removed localities likely to represent *B. bifarius* based on expected distributions (Ghisbain et al., 2020) and by limiting the spatial extent to Longitude −150 to −110 decimal degrees and Latitude 35 to 65 decimal degrees. We only retained observations that had specific descriptive locality information for the record (e.g., at a finer resolution than county) to minimize georeferencing inaccuracy.

We obtained current and historical climate data (BIOCLIM variables, e.g., Hijmans et al., 2005) from ‘rpaleoclim’ 0.9 (Brown et al., 2018). We focused on variables for current, last glacial maximum (LGM, ~20,000 ybp), and last interglacial (LIG, ~130,000 ybp) time periods (Karger et al., 2017, 2020, 2021; Otto‐Bliesner et al., 2006). Given the scale of the study, we used a relatively coarse resolution of 10 arc minutes. We reduced highly correlated variables using hierarchical clustering (hclust in R) and selecting one variable from five cluster groups at a threshold correlation of 0.8: BIO1 (Annual Mean Temperature), BIO8 (Mean Temperature of Wettest Quarter), BIO12 (Annual Precipitation), BIO15 (Precipitation Seasonality), BIO18 (Precipitation of Warmest Quarter). Maps use the WGS84 longitude/latitude coordinate reference system.

We used the maxent version implemented in the R package ‘maxnet’ 0.14 (Phillips, 2017). We used ‘SDMtune’ 1.1.5 (Vignali et al., 2020) to train and refine models. We first filtered the data to reduce spatial bias by removing points within two raster cells of each other (Kiedrzyński et al., 2017). This resulted in a filtered data set of 483 presence points. We randomly split these into 80% training (*N* = 386) and 20% testing (*N* = 97) points. Although the data remain more abundant in the contiguous USA and southern Canada, filtered occurrence records were much more even and largely represent the known distribution of the species (e.g., Stephen, 1957; Thorp et al., 1983). We randomly sampled 10,000 points as background observations and extracted predictor values at each presence and background point using ‘raster’ 3.5–2 (Hijmans, 2020). We trained initial models using the ‘SDMtune’ train function. We used the ‘SDMtune’ optimize Model function to generate models from training data with different combinations of regularization multiplier values (0.5–10) and linear, quadradic, product, and hinge feature classes, and compared the resulting models using AICc. The best model included a regularization multiplier of 1.0 and linear and hinge feature classes. We trained models using selected parameters, plotted the receiver operating characteristic (ROC) curves, and calculated the area under the ROC curve (AUC) for test and training data sets. As a final evaluation of model performance, we used k‐fold cross‐validation using randomly generated sample folds (k = 5) and folds generated using “checkerboard” spatial partitioning (checkerboard1 function, aggregation factor = 10) (Radosavljevic & Anderson, 2014) from the ‘ENMeval’ 2.0.1 package (Kass et al., 2021). Cross‐validation models were trained using the ‘maxnet’ algorithm (called with ‘SDMtune’) and evaluated with AUC (test AUC calculated using mean test AUC across folds). Final models were constructed using the selected tuning options and were spatially and temporally projected onto the current, LGM, and LIG predictor rasters with the default maxent clog‐log 0‐to‐1 scaling.

To evaluate how heterozygosity and the PSMC‐inferred changes in population size may relate to changes in habitat suitability between current and LGM landscapes, we first calculated the “bottleneck size” for each genome as the difference between maximum preglacial *N*_e_ and minimum glacial period *N*_e_ from PSMC (see Figure S1). Next, we calculated a metric of “climatic stability” as the difference in predicted suitability between current and LGM Maxent rasters (positive values indicate less suitability at the LGM, negative values indicate greater suitability at LGM) and extracted values for each locality. Given the similarity of current and LIG maps, we only calculated the current‐LGM difference; changes in suitability at genotyped localities between LGM and today can be interpreted as sites that fluctuate in abiotic suitability given the relative symmetry for LIG‐LGM. As a second metric of stability, we evaluated the proximity of sample sites from areas that would have been suitable during the LGM (the “minimum distance to suitable LGM point”, Figure S1). We determined this distance using the distGeo function in the ‘geosphere’ 1.5–14 R package (Hijmans, 2019) (default WGS84 ellipsoid) to calculate the minimum geographic distance from each sample site to an LGM model raster cell with a predicted suitability ≥ the mean suitability of sites in the current species distribution model (≥ 0.882). Distances were log‐transformed.

We tested the effects of change in suitability at each sample site or distance to the nearest suitable LGM point on both heterozygosity and PSMC‐bottleneck size using lme4 linear mixed effects models, specifying locality as a random effect. The San Juan Island genome was excluded because the population decline persisted and became most severe after the LGM (see Results) and determining the *N*_e_ minimum attributed to the glacial maximum vs changes following glaciation was challenging, as was determining how to incorporate the effects of discrete isolation by water.

## 3 RESULTS

### 3.1 Diversity patterns

PCA revealed clustering consistent with a south‐to‐north gradient in relatedness along PC1 and the San Juan Island genotype separating along PC2 (Figure 2) and sample clustering indicates the regional population groupings should be suitable for coarse comparisons. Diversity levels were significantly different among regions (likelihood ratio test against the model without Region effect: χ^2^ = 261.7, d.f. = 2, *p* < .001; delta‐AIC = −257.7), being highest in northern populations (θ_π_ = 0.0032 per site), intermediate in the mid‐range region (θ_π_ = 0.0030 per site) and lowest in southern populations (θ_π_ = 0.0029 per site), consistent with previous observations (Jackson et al., 2018). Individual heterozygosity showed similar increases from south‐to‐north [linear mixed model: heterozygosity ~ latitude + (1|site), latitude effect *R* ^*2*^_Marginal_ = 0.93, *t* = 16.96, Satterthwaite d.f. = 12.41, *p* < 0.001], except for the San Juan Island genome, which had markedly reduced heterozygosity despite its northern provenance (Figure 2). *F*_ST_ showed the expected weak isolation by distance (Jackson et al., 2018) (south vs mid‐range = 0.026, mid‐range vs north = 0.036, south vs. north = 0.05).

### 3.2 Demographic inferences

#### 3.2.1 PSMC

Shapes of demographic histories were consistent across the 42 moderate (>18x) coverage genomes, suggesting that populations across the Sierra‐Cascades region have been affected by similar species‐wide events (Figure 1b). Nonetheless, curves suggesting a common ancient expansion starting after ~500,000–1 million ybp diverged in maximum postexpansion *N*_e_, with a larger *N*_e_ inferred in northern genotypes compared with mid‐range and southern *N*_e_ between 90,000–150,000 ybp (Figure 1b). Following this peak, PSMC inferred declines during the last glacial period; however, the relative bottleneck in northern populations was much more substantial, which led to similar *N*_e_ among regions during this period. Finally, nearly all mainland populations showed a recent expansion starting ~10,000–30,000 ybp. Contemporary *N*_e_ varied substantially during the most recent time intervals, but most samples showed at least a partial *N*_e_ recovery and recent expansion signatures were stronger in most northern samples (Figure 1b).

Inferences for the high coverage genomes from northern and southern regions were essentially the same as moderate coverage samples (Figure 1c), suggesting that differences in PSMC trajectories were robust to sequencing depth. There was no overlap in bootstraps among samples except for some convergence near the LGM, again suggesting that differences inferred in the moderate coverage genomes were robust. Despite its northern provenance, the high coverage sample from San Juan Island had a historical trajectory similar to the southern genomes, except for the most recent interval that indicated a severe and well‐supported recent bottleneck after 12,000–13,000 ybp (Figure 1c). Inferences were similar when variants were determined using the mappability‐masked reference genome (Figure S2), indicating that mappability was not a major factor.

#### 3.2.2 MSMC2

Major inferences about regional variation in long‐term population sizes were generally supported by MSMC2 (Figure S3). The historical expansion, a late Pleistocene bottleneck, and some degree of postglacial recovery paralleled PSMC. Likewise, the expansion peak *N*_e_ and late Pleistocene bottleneck size were larger in northern populations, with the magnitudes of both declining toward the south. Population *N*_e_ trajectories generally matched the individual‐genome PSMC plots, both for estimates of *N*_e_ and the timing of size changes (Figure S3).

#### 3.2.3 Parallels between summary statistics and SMC models

**FIGURE 3:**
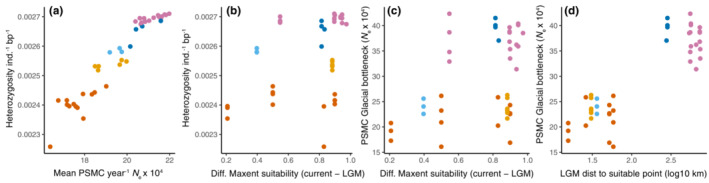
Correlation between mean *N*_e_ over PSMC time intervals and individual heterozygosity for all moderate coverage mainland genomes for *B. vancouverensis* in CA, OR, and WA, USA (a). Relationships between niche stability at each genome coordinate (difference between current maxent suitability and LGM maxent suitability) and heterozygosity (b), niche stability and the size of the PSMC‐inferred glacial period bottleneck (c), and the minimum distance from each sampling coordinate to a pixel with suitable LGM values (suitability values equivalent to the mean current suitability of genome samples shown) and the size of the PSMC‐inferred glacial bottleneck (d). See Figure S1 for a schematic illustrating some of these metrics.

Demographic modeling was consistent with summary statistics from ANGSD in terms of diversity and possible nonequilibrium dynamics. Despite differences in the magnitude of *N*_e_ fluctuations, the mean *N*_e_ over time was greatest in the north and lowest in the south, and mean *N*_e_ was highly correlated with individual heterozygosity, as would be expected given the theoretical relationship between *N*_e_ and heterozygosity (Pearson's *r* = 0.96, *p* < .001; Figure 3a). The downsampled SFS were consistent with the signatures of expansion from PSMC inferences (Figure S4). The number of variants estimated decreased from north to south, with approximately 2.7, 2.5, and 2.3 M variant sites estimated in northern, mid‐range, and southern regions, respectively, but northern populations had the largest proportion of rare variants (i.e., singletons, doubletons, tripletons) and southern populations had the smallest proportion of these rare variants. Likewise, estimates of Tajima's D across scaffolds were most negative in the north (D = −0.62), followed by less negative values in mid‐range (D = −0.19) and southern (D = −0.18) regions.

### 3.3 Distribution models

The final Maxent model had a training and test AUC = 0.86 (Figure S5), and both random and checkerboard spatial cross‐validation also produced AUCs in the 0.85–0.86 range for training and test data. Models from spatially‐filtered observations performed suitably even with some sampling bias toward the southern part of the species range, and the map corresponds with prior models and range maps (Cameron et al., 2011; Thorp et al., 1983). The three models (current, LGM, LIG, Figure 1a) showed fluctuations in the suitable area over time that may explain both species‐wide population trajectories and divergent trajectories in different regions. Present‐day *B. vancouverensis* occupy most mid‐ to high‐elevation montane regions of western North America, Vancouver Island, islands off the coast of Washington (e.g., San Juan Island), and extending north through western Canada and into Alaska. At the LGM, the predicted range contracted everywhere but with most of the northern range becoming unsuitable owing to glacial advance and populations largely restricted to south of present‐day Canada. Exceptions for our northern region include some Oregon locations and regions around Vancouver, BC that maintained a moderate degree of environmental suitability but were isolated. In montane areas, populations would have occupied lower elevations. Mid‐range populations (northern California/southern Oregon) were largely stable, and although the highest elevation southern region sites would have been unsuitable, extensive areas with highly suitable climates would have been available in adjacent lower elevations throughout California. LIG estimates were similar to the present day, consistent with the period of *N*_e_ expansion.

In contrast to expectations for a stability‐diversity hypothesis, heterozygosity was inversely related to the difference in raster suitability between time points (Figure 3b), suggesting that less diverse sites are more climatically stable, although this was not significant (Table S3). There was no significant relationship between the difference in suitability at each locality with the PSMC‐bottleneck size during the last glaciation (Figure 3c; Table S3a). The clearest relationship detected was for the interglacial‐to‐LGM bottleneck size with the minimum distance to a suitable LGM point (Figure 3d, Table S3b): Larger Pleistocene bottlenecks were evident in northern genomes that are more distant from areas with average suitability during the last glacial period. The distance results remained significant when the mean Maxent suitability for all SDM presence coordinates (0.609) was used as a more conservative threshold for minimum distance calculations, rather than the more stringent threshold from genomic sampling sites (0.882) (Table S3c). Given that heterozygosity is greatest in the north and these localities are most distant from suitable LGM raster cells, current heterozygosity was not associated with the distance‐based measure of climatic range stability.

## 4 DISCUSSION

Combining temporal coalescent analyses from modest genome resequencing with paleo‐SDMs proved useful for understanding variation in contemporary genetic diversity in *B. vancouverensis*. Our objective was to better understand north‐to‐south gradients in genetic diversity throughout the Sierra‐Cascade Mountain region of the western USA and to test whether such differences may be explained by long‐term evolutionary processes or might require a more recent demographic explanation. Analyses suggest that all populations experienced fluctuations in *N*_e_ alongside major range shifts. However, variation in genetic diversity can be explained by divergent population histories impacting the degree of these fluctuations, with more diverse contemporary populations exhibiting signatures of elevated *N*_e_ throughout the late Pleistocene, particularly during periods of expansion when the species range was more continuous.

SMC models suggest that *B. vancouverensis* populations fluctuate at the same temporal scales throughout the latitude‐altitude cline examined in this study. It is unsurprising that Quaternary climate dynamics drive concurrent demographic and genetic dynamics (Hewitt, 2000; Shafer et al., 2010), but it is interesting that the magnitudes of fluctuations differ across populations corresponding to the distribution of contemporary genetic variation. Using temporal species distribution models, we examined possible explanations for patterns of diversity and the magnitude of population size fluctuations (Carnaval et al., 2009; Koch et al., 2018). Studies that have investigated the relationship between Quaternary niche stability and genetic diversity have often found that regions with high stability harbor the retention of genetic lineages over time (Carnaval et al., 2009; Vasconcellos et al., 2019), but in other species, including other North American *Bombus*, the inverse relationship has been observed (Koch et al., 2018). Most sites in the *B. vancouverensis* range experienced a decline in habitat suitability at the LGM compared with today and the LIG. But in contrast to expectations under a hypothesis of greater stability driving retention of genetic variation, high‐diversity northern populations experienced the largest loss in LGM suitability. Also somewhat counterintuitively given patterns of contemporary variation, northern genomes experienced much more substantial *N*_e_ reductions through the last glaciation than less diverse southern genomes. Bottleneck magnitudes were larger in the northern populations that are more distant to locations that would have been suitable in the LGM, indicating that these populations would have had to disperse farther to track shifting climates. Although the distance to the LGM location with mean suitability of contemporary sites is not a perfect metric, as sites with below‐average suitability could still have been occupied during the LGM, the strong relationship with PSMC‐bottleneck size suggests it is a reasonable proxy to capture the overall greater habitat loss leading to a larger bottleneck in northern populations during this time.

We hypothesize that the counterintuitive inverse results relating to genetic diversity, historical bottleneck size, and niche stability are driven by the much greater *N*_e_ of northern populations during climatic periods when they expand to become larger and more well‐connected to other parts of the range (e.g., LIG and today), while southern populations remain relatively isolated at high elevations even during favorable periods. Although most populations occupy areas that would have been unsuitable during the LGM, southern populations would have had less genetic variation to lose at this time owing to the weaker interglacial expansion. Furthermore, the southern *B. vancouverensis* range did not suffer a regional collapse of suitable habitats at the LGM; high‐elevation populations would have only had to shift to nearby lower elevations via short distance dispersal to stay within their niche, contributing to greater stability and more tempered bottleneck signatures. By contrast, the northern range largely disappeared due to the presence of ice sheets (Shafer et al., 2010), alongside the most suitable connections to eastern *B. vancouverensis* populations (Lozier et al., 2013, 2016). Given that northern populations were likely much larger and well connected to other parts of the *B. vancouverensis* range prior to the LGM, as they are today, such habitat loss would have had disproportionately large effects on *N*_e_. Conversely, connections remade after the LGM could supplement *N*_e_ via colonization from multiple founding populations (Ortego et al., 2015). Our results thus suggest that booms in heterozygosity from massive spatial and *N*_e_ expansions in the north during favorable climates likely offset any busts from contractions in less favorable periods.

Focusing on bumble bees, a recent analysis of *Bombus huntii* in western North America (Koch et al., 2018) found nearly identical relationships for genetic diversity and Pleistocene niche stability, with northern populations exhibiting both relatively high contemporary heterozygosity and relatively low predicted LGM niche suitability compared with southern parts of the species range. Interglacial and postglacial increases in *N*_e_ for species like *B. vancouverensis* and *B. huntii* contrasts with some expectations for cold‐adapted bumble bees in other regions, which can expand their ranges during glacial periods and contract to interglacial refugia in warm periods (Hewitt, 2011; Martinet et al., 2018; Williams et al., 2018). However, models of reduced population size during glacial periods have also been supported for montane insects of western North America (Schoville & Roderick, 2009). Thus, diversity in bumble bees and other widespread montane species is likely determined by the degree of habitat stability alongside the arrangement of that habitat that can facilitate or constrain population expansion in particular regions in particular climates (Lee et al., 2019; Williams et al., 2018, 2022). For many cold‐adapted North American *Bombus* species, southern parts of species ranges tend to be restricted to relatively high elevations. While such parts of the range may be relatively stable as populations can simply shift down and upslope to nearby areas with climate fluctuations (Lee et al., 2019), maximum habitable areas, and consequently *Ne*, may be reduced compared with high‐latitude populations. Population dynamics should vary in parts of the globe with different orientations of major mountains, and it would be interesting to compare our results with other regions.

The potential connections of northern populations to other parts of the *B. vancouverensis* range discussed above raises an important caveat related to SMC models in general. Although employing multiple SMC methods can strengthen inferences, like any method results are sensitive to the assumed model (reviewed in Mather et al., 2020). For example, periodic changes in migration and population structure like that discussed above can also influence the coalescent *N*_e_ and the shape of SMC‐inferred trajectories without major changes in true population size (Chikhi et al., 2018; Mazet et al., 2016). By comparing many individual genomes across a species range, consistent variation in the magnitude or timing of population size peaks and troughs still provide evidence for historical demographic changes influencing contemporary diversity. Because genomes from southern populations show persistently low *N*_e_ compared with northern genomes, we infer that processes during the Quaternary established conditions that favor the maintenance of relatively low genetic diversity without needing to invoke strong effects of very recent (i.e., Holocene, Anthropocene) processes. The correlations with predicted habitable area from SDMs also provide support for the reliability of the SMC models; however, because we expect changes in population structure over time, some inferred differences could be due to migration rates rather than a simple reflection of the numbers of individuals alone. Another potential concern is that, while temporal SDMs have been commonly used for understanding patterns of genetic diversity, these models assume that a species niche is conserved through time, with no temporal adaptation to changing conditions that might allow persistence in regions with low predicted suitability based on contemporary occurrences. Local adaptation may be present across the *B. vancouverensis* range (Heraghty et al., 2022), and it is possible that both the SDM projection at the LGM is pessimistic and that *N*_e_ trajectories could be altered by localized selection signatures in the genome, both of which could influence our conclusions. The temporal concordance of range size and *N*_e_ dynamics provide some support for conclusions from each method, but the development of methods for coalescent inference and niche modeling that incorporate space, time, and natural selection would greatly assist our understanding of historical demography in *B. vancouverensis* and most other species (Chikhi et al., 2018).

We also cannot unambiguously state that recent demographic changes are not influencing *B. vancouverensis* alongside Quaternary fluctuations because SMC methods tend to perform relatively poorly at contemporary scales. However, the available San Juan Island genome (Ghisbain et al., 2020) is a useful comparison to mainland samples by providing something of positive control for the speed with which SMC models can track diversity loss when isolation is more extreme. We expected that recent changes could be detectable in bumble bees, even for “long‐term” SMC estimators, due to their annual generations. Indeed, unlike other samples, the San Juan Island genome indicated a striking contemporary *N*_e_ decline. Although predicted suitability fluctuated over time and contemporary suitability is high, the island's isolation following the glacial retreat likely would have led to conditions favoring significant genetic isolation (Lozier et al., 2011). Strong genetic signatures of isolation are common in bumble bees on coastal islands (Goulson et al., 2011; Jha, 2015; Lozier et al., 2011), providing a model of habitat fragmentation effects that may become common under climate change (Frankham, 1996). If lower elevational range limits erode under climate change (Kerr et al., 2015), populations in southern parts of the *B. vancouverensis* range will likely be pushed upslope and become increasingly isolated (Parmesan, 2006; Urban, 2018). This “sky island” isolation could begin to mimic the signatures observed on true islands (Epps et al., 2006; McDonald & Brown, 1992; Urban, 2018), even if long‐term range dynamics drive most of the current variation in heterozygosity. Other works in bumble bees suggest Arctic populations may be at even greater risk than montane populations under climate change (Lee et al., 2019), so high‐latitude *B. vancouverensis* populations are also potentially at risk despite their currently greater habitable area and genetic diversity. Additional work to understand demographic changes within the last century and monitor future genetic diversity will become important components of montane *Bombus* conservation.

In conclusion, demographic analyses suggest that populations of montane bumble bees may rapidly respond and maintain genetic diversity following climate‐associated range perturbations and that gradients in diversity can be explained to a large extent by differences in these historical fluctuations. Some caution is thus likely warranted when attempting to link patterns of contemporary *Bombus* genetic diversity to human activities. To some extent, such patterns are encouraging for the long‐term genetic health of bumble bee populations. However, there is reason to expect that future climate change will drive montane pollinator populations further upslope (Kerr et al., 2015), and given that long‐term *N*_e_ and heterozygosity appear constrained in high‐elevation populations, warming temperatures may further erode habitable areas and population connectivity in this region. The distinct postglacial bottleneck inferred for the only truly insular sample suggests that population isolation is a concern for the erosion of genetic diversity when sufficiently strong and persistent. Thus, caution is also warranted for predicting future *N*_e_ stability from relatively large *N*_e_′s of the past. Additional sequencing of *Bombus* from other fragmented populations would provide insights into the temporal dynamics and consistency of reduced population sizes in insular habitats like islands, mountaintops, or disturbed habitat patches.

## AUTHOR CONTRIBUTIONS

**Jeffrey D Lozier:** Conceptualization (lead); data curation (lead); formal analysis (equal); funding acquisition (equal); investigation (equal); methodology (equal); project administration (lead); visualization (lead); writing – original draft (lead). **Jamie Strange:** Funding acquisition (equal); investigation (equal); resources (equal); writing – review and editing (equal). **Sam D Heraghty:** Data curation (equal); formal analysis (equal); investigation (equal); writing – review and editing (equal).

## FUNDING INFORMATION

US National Science Foundation DEB 1457645, URoL 1921585.

## CONFLICT OF INTEREST STATEMENT

Neither I nor my co‐authors have a conflict of interest. No materials should require permission to reproduce.

## Supporting information


Appendix S1

## Data Availability

All sequence data used in the study have been deposited at the National Center for Biotechnology Information Sequence Read Archive (see Appendix S1, Table S2 for accessions). Genetic data outputs, coordinate data from GBIF.org, filtered records and random background points with climate data, stability metric calculations, and example code are deposited on DRYAD (10.5061/dryad.dbrv15f4m). https://doi.org/10.5061/dryad.dbrv15f4m.

## References

[ece39778-bib-0001] Bates, D. , Mächler, M. , Bolker, B. , & Walker, S. (2015). Fitting linear mixed‐effects models using lme4. Journal of Statistical Software, 67(1), 1–48. 10.18637/jss.v067.i01

[ece39778-bib-0002] Biella, P. , Bogliani, G. , Cornalba, M. , Manino, A. , Neumayer, J. , Porporato, M. , Rasmont, P. , & Milanesi, P. (2017). Distribution patterns of the cold adapted bumblebee *Bombus alpinus* in the Alps and hints of an uphill shift (Insecta: Hymenoptera: Apidae). Journal of Insect Conservation, 21(2), 357–366. 10.1007/s10841-017-9983-1

[ece39778-bib-0003] Broad Institute . (2019). Picard . Retrieved from. http://broadinstitute.github.io/picard/

[ece39778-bib-0004] Brown, J. L. , Hill, D. J. , Dolan, A. M. , Carnaval, A. C. , & Haywood, A. M. (2018). Paleoclim, high spatial resolution paleoclimate surfaces for global land areas. Scientific Data, 5, 1–9. 10.1038/sdata.2018.254 30422125 PMC6233254

[ece39778-bib-0005] Bushnell, B. (2020). BBMap . Retrieved from. http://sourceforge.net/, projects/bbmap/

[ece39778-bib-0006] Cameron, S. A. , Lozier, J. D. , Strange, J. P. , Koch, J. B. , Cordes, N. , Solter, L. F. , & Griswold, T. L. (2011). Patterns of widespread decline in north American bumble bees. Proceedings of the National Academy of Sciences, 108(2), 662–667. 10.1073/pnas.1014743108 PMC3021065 21199943

[ece39778-bib-0007] Cameron, S. A. , & Sadd, B. M. (2020). Global trends in bumble bee health. Annual Review of Entomology, 65(1), 209–232. 10.1146/annurev-ento-011118-111847 31610137

[ece39778-bib-0008] Canadian Biodiversity Information Facility . (2021). Bombus of Canada . Occurrence Dataset. 10.15468/ip9oon

[ece39778-bib-0009] Carnaval, A. C. , Hickerson, M. J. , Haddad, C. F. B. , Rodrigues, M. T. , & Moritz, C. (2009). Stability predicts genetic diversity in the Brazilian Atlantic forest hotspot. Science, 323(5915), 785–789. 10.1126/science.1166955 19197066

[ece39778-bib-0010] Chikhi, L. , Rodríguez, W. , Grusea, S. , Santos, P. , Boitard, S. , & Mazet, O. (2018). The IICR (Inverse Instantaneous Coalescence Rate) as a summary of genomic diversity: Insights into demographic inference and model choice. Heredity, 120(1), 13–24. 10.1038/s41437-017-0005-6 29234166 PMC5837117

[ece39778-bib-0011] Christmas, M. J. , Jones, J. C. , Olsson, A. , Wallerman, O. , Bunikis, I. , Kierczak, M. , Peona, V. , Whitley, K. M. , Larva, T. , Suh, A. , Miller‐Struttmann, N. E. , Geib, J. C. , & Webster, M. T. (2021). Genetic barriers to historical gene flow between cryptic species of alpine bumblebees revealed by comparative population genomics. Molecular Biology and Evolution, 38(8), 3126–3143. 10.1093/molbev/msab086 33823537 PMC8321533

[ece39778-bib-0012] Dellicour, S. , Kastally, C. , Varela, S. , Michez, D. , Rasmont, P. , Mardulyn, P. , & Lecocq, T. (2017). Ecological niche modelling and coalescent simulations to explore the recent geographical range history of five widespread bumblebee species in Europe. Journal of Biogeography, 44(1), 39–50. 10.1111/jbi.12748

[ece39778-bib-0013] Dmitriev, D. (2015). Illinois natural history survey insect collection. Illinois Natural History Survey. Occurrence Dataset. 10.15468/eol0pe

[ece39778-bib-0014] Elith, J. , Phillips, S. J. , Hastie, T. , Dudík, M. , Chee, Y. E. , & Yates, C. J. (2011). A statistical explanation of MaxEnt for ecologists. Diversity and Distributions, 17(1), 43–57. 10.1111/j.1472-4642.2010.00725.x

[ece39778-bib-0015] Ellis, J. S. , Knight, M. E. , Darvill, B. , & Goulson, D. (2006). Extremely low effective population sizes, genetic structuring and reduced genetic diversity in a threatened bumblebee species, *Bombus sylvarum* (Hymenoptera: Apidae). Molecular Ecology, 15(14), 4375–4386. 10.1111/j.1365-294X.2006.03121.x 17107471

[ece39778-bib-0016] Epps, C. W. , Palsbøll, P. J. , Wehausen, J. D. , Roderick, G. K. , & McCullough, D. R. (2006). Elevation and connectivity define genetic refugia for mountain sheep as climate warms. Molecular Ecology, 15(14), 4295–4302. 10.1111/j.1365-294X.2006.03103.x 17107466

[ece39778-bib-0017] Foote, A. D. , Hooper, R. , Alexander, A. , Baird, R. W. , Baker, C. S. , Ballance, L. , Barlow, J. , Brownlow, A. , Collins, T. , Constantine, R. , Dalla Rosa, L. , Davison, N. J. , Durban, J. W. , Esteban, R. , Excoffier, L. , Martin, S. L. F. , Forney, K. A. , Gerrodette, T. , Gilbert, M. T. P. , … Morin, P. A. (2021). Runs of homozygosity in killer whale genomes provide a global record of demographic histories. Molecular Ecology, 30(23), 6162–6177. 10.1111/mec.16137 34416064

[ece39778-bib-0018] Frankham, R. (1996). Relationship of genetic variation to population size in wildlife. Conservation Biology, 10(6), 1500–1508. 10.1046/j.1523-1739.1996.10061500.x

[ece39778-bib-0019] Gall, L. (2021). Entomology division, Yale Peabody Museum. Yale University Peabody Museum. Occurrence Dataset. 10.15468/95waq3.

[ece39778-bib-0020] Garrick, R. C. , Hyseni, C. , Arantes, Í. C. , Zachos, L. G. , Zee, P. C. , & Oliver, J. C. (2021). Is phylogeographic congruence predicted by historical habitat stability, or ecological co‐associations? Insect Systematics and Diversity, 5(5), 7. 10.1093/isd/ixab018

[ece39778-bib-0021] Ghisbain, G. , Lozier, J. D. , Rahman, S. R. , Ezray, B. D. , Tian, L. , Ulmer, J. M. , Heraghty, S. D. , Strange, J. P. , Rasmont, P. , & Hines, H. M. (2020). Substantial genetic divergence and lack of recent gene flow support cryptic speciation in a colour polymorphic bumble bee (*Bombus bifarius*) species complex. Systematic Entomology, 45(3), 635–652. 10.1111/syen.12419

[ece39778-bib-0022] Goulson, D. (2010). Bumblebees; their behaviour, ecology and conservation. Oxford University Press.

[ece39778-bib-0023] Goulson, D. , Kaden, J. C. , Lepais, O. , Lye, G. C. , & Darvill, B. (2011). Population structure, dispersal and colonization history of the garden bumblebee *Bombus hortorum* in the Western isles of Scotland. Conservation Genetics, 12(4), 867–879. 10.1007/s10592-011-0190-4

[ece39778-bib-0024] Goulson, D. , Lye, G. C. C. , & Darvill, B. (2008). Decline and conservation of bumble bees. Annual Review of Entomology, 53(1), 191–208. 10.1146/annurev.ento.53.103106.093454 17803456

[ece39778-bib-0025] Heinrich, B. (2004). Bumblebee economics. Harvard University Press.

[ece39778-bib-0026] Heraghty, S. D. , Rahman, S. R. , Jackson, J. M. , & Lozier, J. D. (2022). Whole genome sequencing reveals the structure of environment associated divergence in a broadly distributed montane bumble bee, *Bombus vancouverensis* . Insect Systematics and Diversity, 6(5), 5.

[ece39778-bib-0027] Heraghty, S. D. , Sutton, J. M. , Pimsler, M. L. , Fierst, J. L. , Strange, J. P. , & Lozier, J. D. (2020). De novo genome assemblies for three north American bumble bee species: *Bombus bifarius*, *Bombus vancouverensis*, and *Bombus vosnesenskii* . G3: Genes, Genomes, Genetics, 10(8), 2585–2592. 10.1534/g3.120.401437 32586847 PMC7407468

[ece39778-bib-0028] Hewitt, G. M. (2000). The genetic legacy of the quaternary ice ages. Nature, 405, 907–913. 10879524 10.1038/35016000

[ece39778-bib-0029] Hewitt, G. M. (2011). Quaternary phylogeography: The roots of hybrid zones. Genetica, 139(5), 617–638. 10.1007/s10709-011-9547-3 21234647

[ece39778-bib-0030] Hijmans, R. J. (2019). Geosphere: Spherical trigonometry . Retrieved from. https://cran.r‐project.org/package=geosphere

[ece39778-bib-0031] Hijmans, R. J. (2020). Raster: Geographic data analysis and modeling . Retrieved from. https://cran.r‐project.org/package=raster

[ece39778-bib-0032] Hijmans, R. J. , Cameron, S. E. , Parra, J. L. , Jones, P. G. , & Jarvis, A. (2005). Very high resolution interpolated climate surfaces for global land areas. International Journal of Climatology, 25(15), 1965–1978. 10.1002/joc.1276

[ece39778-bib-0033] Holderegger, R. , & Wagner, H. H. (2008). Landscape genetics. Bioscience, 58(3), 199–207. 10.1641/B580306

[ece39778-bib-0034] Ikerd, H. (2019). Bee biology and systematics laboratory . USDA‐ARS Pollinating Insect‐Biology, Management, Systematics Research. Occurrence dataset. 10.15468/anyror

[ece39778-bib-0035] Iserbyt, S. , & Rasmont, P. (2012). The effect of climatic variation on abundance and diversity of bumblebees: A ten years survey in a mountain hotspot. Annales de La Société Entomologique de France, 48(3–4), 414–426. 10.1080/00379271.2012.10697775

[ece39778-bib-0036] Jackson, J. M. , Pimsler, M. L. , Oyen, K. J. , Koch‐Uhuad, J. B. , Herndon, J. D. , Strange, J. P. , Dillon, M. E. , & Lozier, J. D. (2018). Distance, elevation and environment as drivers of diversity and divergence in bumble bees across latitude and altitude. Molecular Ecology, 27(14), 2926–2942. 10.1111/mec.14735 29862596

[ece39778-bib-0037] Jackson, J. M. , Pimsler, M. L. , Oyen, K. J. , Strange, J. P. , Dillon, M. E. , & Lozier, J. D. (2020). Local adaptation across a complex bioclimatic landscape in two montane bumble bee species. Molecular Ecology, 29(5), 920–939. 10.1111/mec.15376 32031739

[ece39778-bib-0038] Jha, S. (2015). Contemporary human‐altered landscapes and oceanic barriers reduce bumble bee gene flow. Molecular Ecology, 24(5), 993–1006. 10.1111/mec.13090 25626470

[ece39778-bib-0039] Karger, D. N. , Conrad, O. , Böhner, J. , Kawohl, T. , Kreft, H. , Soria‐Auza, R. W. , Zimmermann, N. E. , Linder, H. P. , & Kessler, M. (2017). Climatologies at high resolution for the earth's land surface areas. Scientific Data, 4, 1–20. 10.1038/sdata.2017.122 PMC5584396 28872642

[ece39778-bib-0040] Karger, D. N. , Nobis, M. P. , Normand, S. , Graham, C. H. , & Zimmermann, N. E. (2021). CHELSA‐TraCE21k v1.0. Downscaled transient temperature and precipitation data since the last glacial maximum. Climate of the Past Discussions, 1–27. 10.5194/cp-2021-30

[ece39778-bib-0041] Karger, D. N. , Schmatz, D. R. , Dettling, G. , & Zimmermann, N. E. (2020). High‐resolution monthly precipitation and temperature time series from 2006 to 2100. Scientific Data, 7(1), 1–10. 10.1038/s41597-020-00587-y 32703947 PMC7378208

[ece39778-bib-0042] Kass, J. M. , Muscarella, R. , Galante, P. J. , Bohl, C. L. , Pinilla‐Buitrago, G. E. , Boria, R. A. , Soley‐Guardia, M. , & Anderson, R. P. (2021). ENMeval 2.0: Redesigned for customizable and reproducible modeling of species' niches and distributions. Methods in Ecology and Evolution, 12, 1602–1608. 10.1111/2041-210X.13628

[ece39778-bib-0043] Kerr, J. T. , Pindar, A. , Galpern, P. , Packer, L. , Potts, S. G. , Roberts, S. M. , Rasmont, P. , Schweiger, O. , Colla, S. R. , Richardson, L. L. , Wagner, D. L. , Gall, L. F. , Sikes, D. S. , & Pantoja, A. (2015). Climate change impacts on bumblebees converge across continents. Science, 349(6244), 177–180. 10.1126/science.aaa7031 26160945

[ece39778-bib-0044] Kiedrzyński, M. , Zielińska, K. M. , Rewicz, A. , & Kiedrzyńska, E. (2017). Habitat and spatial thinning improve the maxent models performed with incomplete data. Journal of Geophysical Research – Biogeosciences, 122(6), 1359–1370. 10.1002/2016JG003629

[ece39778-bib-0045] Koch, J. B. , Lozier, J. , Strange, J. P. , Ikerd, H. , Griswold, T. , Cordes, N. , & Cameron, S. A. (2015). USBombus, a database of contemporary survey data for north American bumble bees (Hymenoptera, Apidae, Bombus) distributed in the United States. Biodiversity Data Journal, 3(1), e6833. 10.3897/BDJ.3.e6833 PMC4698456 26751762

[ece39778-bib-0046] Koch, J. B. , Vandame, R. , Mérida‐Rivas, J. , Sagot, P. , & Strange, J. (2018). Quaternary climate instability is correlated with patterns of population genetic variability in *Bombus huntii* . Ecology and Evolution, 8(16), 7849–7864. 10.1002/ece3.4294 30250668 PMC6145020

[ece39778-bib-0047] Korneliussen, T. S. , Albrechtsen, A. , & Nielsen, R. (2014). ANGSD: Analysis of next generation sequencing data. BMC Bioinformatics, 15(1), 356. 10.1186/s12859-014-0356-4 25420514 PMC4248462

[ece39778-bib-0048] Korneliussen, T. S. , Moltke, I. , Albrechtsen, A. , & Nielsen, R. (2013). Calculation of Tajima's D and other neutrality test statistics from low depth next‐generation sequencing data. BMC Bioinformatics, 14, 289. 10.1186/1471-2105-14-289 24088262 PMC4015034

[ece39778-bib-0049] Kuznetsova, A. , Brockhoff, P. B. , & Christensen, R. H. B. (2017). lmerTest package: Tests in linear mixed effects models. Journal of Statistical Software, 82(13), 1–26. 10.18637/jss.v082.i13

[ece39778-bib-0050] Lee, C. K. F. , Williams, P. H. , & Pearson, R. G. (2019). Climate change vulnerability higher in arctic than alpine bumblebees. Frontiers of Biogeography, 11(4), 1–15.

[ece39778-bib-0051] Li, H. (2013). Aligning sequence reads, clone sequences and assembly contigs with BWA‐MEM . 10.48550/arXiv.1303.3997

[ece39778-bib-0052] Li, H. , & Durbin, R. (2011). Inference of human population history from individual whole‐genome sequences. Nature, 475(7357), 493–496. 10.1038/nature10231 21753753 PMC3154645

[ece39778-bib-0053] Li, H. , Handsaker, B. , Wysoker, A. , Fennell, T. , Ruan, J. , Homer, N. , Marth, G. , Abecasis, G. , Durbin, R. , & 1000 Genome Project Data Processing Subgroup . (2009). The sequence alignment/map format and SAMtools. Bioinformatics, 25(16), 2078–2079. 10.1093/bioinformatics/btp352 19505943 PMC2723002

[ece39778-bib-0054] Liu, H. , Jia, Y. , Sun, X. , Tian, D. , Hurst, L. D. , & Yang, S. (2017). Direct determination of the mutation rate in the bumblebee reveals evidence for weak recombination‐associated mutation and an approximate rate constancy in insects. Molecular Biology and Evolution, 34(1), 119–130. 10.1093/molbev/msw226 28007973 PMC5854123

[ece39778-bib-0055] Lozier, J. D. (2014). Revisiting comparisons of genetic diversity in stable and declining species: Assessing genome‐wide polymorphism in north American bumble bees using RAD sequencing. Molecular Ecology, 23(4), 788–801. 10.1111/mec.12636 24351120

[ece39778-bib-0056] Lozier, J. D. , Jackson, J. M. , Dillon, M. E. , & Strange, J. P. (2016). Population genomics of divergence among extreme and intermediate color forms in a polymorphic insect. Ecology and Evolution, 6(4), 1075–1091. 10.1002/ece3.1928 26811748 PMC4722823

[ece39778-bib-0057] Lozier, J. D. , Parsons, Z. M. , Rachoki, L. , Jackson, J. M. , Pimsler, M. L. , Oyen, K. J. , Strange, J. , & Dillon, M. E. (2021). Divergence in body mass, wing loading, and population structure reveals species‐specific and potentially adaptive trait variation across elevations in montane bumble bees. Insect Systematics and Diversity, 5(5), 3. 10.1093/isd/ixab012

[ece39778-bib-0058] Lozier, J. D. , Strange, J. P. , & Koch, J. B. (2013). Landscape heterogeneity predicts gene flow in a widespread polymorphic bumble bee, *Bombus bifarius* (Hymenoptera: Apidae). Conservation Genetics, 14(5), 1099–1110. 10.1007/s10592-013-0498-3

[ece39778-bib-0059] Lozier, J. D. , Strange, J. P. , Stewart, I. J. , & Cameron, S. A. (2011). Patterns of range‐wide genetic variation in six north American bumble bee (Apidae: *Bombus*) species. Molecular Ecology, 20(23), 4870–4888. 10.1111/j.1365-294X.2011.05314.x 22035452

[ece39778-bib-0060] Marshall, L. , Perdijk, F. , Dendoncker, N. , Kunin, W. , Roberts, S. , & Biesmeijer, J. C. (2020). Bumblebees moving up: Shifts in elevation ranges in the Pyrenees over 115 years. Proceedings of the Royal Society B: Biological Sciences, 287(1938), 20202201. 10.1098/rspb.2020.2201 PMC7735265 33171083

[ece39778-bib-0061] Martinet, B. , Dellicour, S. , Ghisbain, G. , Przybyla, K. , Zambra, E. , Lecocq, T. , Boustani, M. , Baghirov, R. , Michez, D. , & Rasmont, P. (2020). Global effects of extreme temperatures on wild bumblebees. Conservation Biology, 35(5), 1507–1518. 10.1111/cobi.13685 33319368

[ece39778-bib-0062] Martinet, B. , Lecocq, T. , Brasero, N. , Biella, P. , UrbanovÁ, K. , ValterovÁ, I. , Cornalba, M. , Gjershaug, J. O. , Michez, D. , & Rasmont, P. (2018). Following the cold: Geographical differentiation between interglacial refugia and speciation in the arcto‐alpine species complex *Bombus monticola* (Hymenoptera: Apidae). Systematic Entomology, 43(1), 200–217. 10.1111/syen.12268

[ece39778-bib-0063] Mather, N. , Traves, S. M. , & Ho, S. Y. W. (2020). A practical introduction to sequentially Markovian coalescent methods for estimating demographic history from genomic data. Ecology and Evolution, 10(1), 579–589. 10.1002/ece3.5888 31988743 PMC6972798

[ece39778-bib-0064] Mazet, O. , Rodríguez, W. , Grusea, S. , Boitard, S. , & Chikhi, L. (2016). On the importance of being structured: Instantaneous coalescence rates and human evolution—Lessons for ancestral population size inference? Heredity, 116(4), 362–371. 10.1038/hdy.2015.104 26647653 PMC4806692

[ece39778-bib-0065] McDonald, K. A. , & Brown, J. H. (1992). Using montane mammals to model extinctions due to global change. Conservation Biology, 6(3), 409–415. 10.1046/j.1523-1739.1992.06030409.x

[ece39778-bib-0066] McKenna, A. , Hanna, M. , Banks, E. , Sivachenko, A. , Cibulskis, K. , Kernytsky, A. , Garimella, K. , Altshuler, D. , Gabriel, S. , Daly, M. , & DePristo, M. (2010). The genome analysis toolkit: A map reduce framework for analyzing next‐generation DNA sequencing data. Genome Research, 20(9), 1297–1303. 20644199 10.1101/gr.107524.110 PMC2928508

[ece39778-bib-0067] McRae, B. H. , & Beier, P. (2007). Circuit theory predicts gene flow in plant and animal populations. Proceedings of the National Academy of Sciences of the United States of America, 104(50), 19885–19890. 10.1073/pnas.0706568104 18056641 PMC2148392

[ece39778-bib-0068] Meisner, J. , & Albrechtsen, A. (2018). Inferring population structure and admixture proportions in low‐depth NGS data. Genetics, 210(2), 719–731. 10.1534/genetics.118.301336 30131346 PMC6216594

[ece39778-bib-0069] Montero‐Mendieta, S. , Tan, K. , Christmas, M. J. , Olsson, A. , Vilà, C. , Wallberg, A. , & Webster, M. T. (2019). The genomic basis of adaptation to high‐altitude habitats in the eastern honey bee (Apis cerana). Molecular Ecology, 28(4), 746–760. 10.1111/mec.14986 30576015

[ece39778-bib-0070] Nadachowska‐Brzyska, K. , Burri, R. , Smeds, L. , & Ellegren, H. (2016). PSMC analysis of effective population sizes in molecular ecology and its application to black‐and‐white *Ficedula* flycatchers. Molecular Ecology, 25(5), 1058–1072. 10.1111/mec.13540 26797914 PMC4793928

[ece39778-bib-0071] Nielsen, R. , Korneliussen, T. , Albrechtsen, A. , Li, Y. , & Wang, J. (2012). SNP calling, genotype calling, and sample allele frequency estimation from new‐generation sequencing data. PLoS One, 7(7), e37558. 10.1371/journal.pone.0037558 22911679 PMC3404070

[ece39778-bib-0072] Orrell, T. (2021). NMNH extant specimen records. Version 1.48 . National Museum of Natural History, Smithsonian Institution. Occurrence dataset. 10.15468/hnhrg3

[ece39778-bib-0073] Ortego, J. , Gugger, P. F. , & Sork, V. L. (2015). Climatically stable landscapes predict patterns of genetic structure and admixture in the Californian canyon live oak. Journal of Biogeography, 42(2), 328–338. 10.1111/jbi.12419

[ece39778-bib-0074] Otto‐Bliesner, B. L. , Marshall, S. J. , Overpeck, J. T. , Miller, G. H. , Hu, A. , & CAPE Last Interglacial Project Members . (2006). Simulating arctic climate warmth and icefield retreat in the last interglaciation. Science, 311(5768), 1751–1753. 10.1126/science.1120808 16556838

[ece39778-bib-0075] Parmesan, C. (2006). Ecological and evolutionary responses to recent climate change. Annual Review of Ecology, Evolution, and Systematics, 37(1), 637–669. 10.1146/annurev.ecolsys.37.091305.110100

[ece39778-bib-0076] Patil, A. B. , & Vijay, N. (2021). Repetitive genomic regions and the inference of demographic history. Heredity, 127(2), 151–166. 10.1038/s41437-021-00443-8 34002046 PMC8322061

[ece39778-bib-0077] Phillips, S. J. (2017). maxnet: Fitting “Maxent” Species Distribution Models with “glmnet” . Retrieved from. https://cran.r‐project.org/package=maxnet

[ece39778-bib-0078] Phillips, S. J. , Anderson, R. P. , & Schapire, R. E. (2006). Maximum entropy modeling of species geographic distributions. Ecological Modelling, 190, 231–259.

[ece39778-bib-0079] Pradervand, J. N. , Pellissier, L. , Randin, C. F. , & Guisan, A. (2014). Functional homogenization of bumblebee communities in alpine landscapes under projected climate change. Climate Change Responses, 1(1), 1–10. 10.1186/s40665-014-0001-5

[ece39778-bib-0080] R Core Team . (2021). R: A language and environment for statistical computing. R Core Team. Retrieved from. https://www.r‐project.org/

[ece39778-bib-0081] Radosavljevic, A. , & Anderson, R. P. (2014). Making better maxent models of species distributions: Complexity, overfitting and evaluation. Journal of Biogeography, 41(4), 629–643. 10.1111/jbi.12227

[ece39778-bib-0082] Rasmont, P. , Franzen, M. , Lecocq, T. , Harpke, A. , Roberts, S. P. M. , Biesmeijer, J. C. , Castro, L. , Cederberg, B. , Dvořák, L. , Fitzpatrick, Ú. , Gonseth, Y. , Haubruge, E. , Mahé, G. , Manino, A. , Michez, D. , Neumayer, J. , Ødegraad, F. , Paukkunen, J. , Pawlikowski, T. , … Schweiger, O. (2015). Climatic risk and distribution atlas of European bumblebees. Pensoft., 10, 1–236.

[ece39778-bib-0083] Rubidge, E. M. , Patton, J. L. , Lim, M. , Burton, A. C. , Brashares, J. S. , & Moritz, C. (2012). Climate‐induced range contraction drives genetic erosion in an alpine mammal. Nature Climate Change, 2(4), 285–288. 10.1038/nclimate1415

[ece39778-bib-0084] Schiffels, S. , & Durbin, R. (2014). Inferring human population size and separation history from multiple genome sequences. Nature Genetics, 46(8), 919–925. 10.1038/ng.3015 24952747 PMC4116295

[ece39778-bib-0085] Schiffels, S. , & Wang, K. (2020). MSMC and MSMC2: The multiple sequentially Markovian coalescent. In J. Y. Dutheil (Ed.), Statistical population genomics (pp. 147–166). Springer US. 10.1007/978-1-0716-0199-0_7 31975167

[ece39778-bib-0086] Schoville, S. D. , & Roderick, G. K. (2009). Alpine biogeography of Parnassian butterflies during quaternary climate cycles in North America. Molecular Ecology, 18(16), 3471–3485. 10.1111/j.1365-294X.2009.04287.x 19659481

[ece39778-bib-0087] Shafer, A. B. A. , Cullingham, C. I. , Côté, S. D. , & Coltman, D. W. (2010). Of glaciers and refugia: A decade of study sheds new light on the phylogeography of northwestern North America. Molecular Ecology, 19(21), 4589–4621. 10.1111/j.1365-294X.2010.04828.x 20849561

[ece39778-bib-0088] Spence, J. P. , Steinrücken, M. , Terhorst, J. , & Song, Y. S. (2018). Inference of population history using coalescent HMMs: Review and outlook. Current Opinion in Genetics and Development, 53, 70–76. 10.1016/j.gde.2018.07.002 30056275 PMC6296859

[ece39778-bib-0089] Stephen, W. P. (1957). Bumble bees of Western America (Hymenoptera: Apoidea). Oregon State University Technical Bulletin, 40, 1–163.

[ece39778-bib-0090] Storfer, A. , Murphy, M. A. , Evans, J. S. , Goldberg, C. S. , Robinson, S. , Spear, S. F. , Dezzani, R. , Delmelle, E. , Vierling, L. , & Waits, L. P. (2007). Putting the “landscape” in landscape genetics. Heredity, 98(3), 128–142. 10.1038/sj.hdy.6800917 17080024

[ece39778-bib-0091] Strange, J. (2019). Patterns of widespread decline in north American bumble bees . USDA‐ARS pollinating insect‐biology, management, systematics research. Occurrence dataset. 10.15468/kjpwz1 PMC3021065 21199943

[ece39778-bib-0092] Strange, J. , Tripodi, A. , & Ikerd, H. (2021). Characterizing bumble bee (Bombus) communities in the United States and assessing a conservation monitoring method. Version 1.5 . USDA‐ARS pollinating insect‐biology, management, systematics research. Occurrence dataset. 10.1002/ece3.4783 PMC6374645 30805140

[ece39778-bib-0093] Struebig, M. J. , Kingston, T. , Petit, E. J. , Le Comber, S. C. , Zubaid, A. , Mohd‐Adnan, A. , & Rossiter, S. J. (2011). Parallel declines in species and genetic diversity in tropical forest fragments. Ecology Letters, 14(6), 582–590. 10.1111/j.1461-0248.2011.01623.x 21564453

[ece39778-bib-0094] Taylor, R. S. , Manseau, M. , Klütsch, C. F. C. , Polfus, J. L. , Steedman, A. , Hervieux, D. , Kelly, A. , Larter, N. C. , Gamberg, M. , Schwantje, H. , & Wilson, P. J. (2021). Population dynamics of caribou shaped by glacial cycles before the last glacial maximum. Molecular Ecology, 30(23), 6121–6143. 10.1111/mec.16166 34482596 PMC9293238

[ece39778-bib-0095] Thorp, R. W. , Horning, D. S. J. , & Dunning, L. L. (1983). Bumble bees and cuckoo bumble bees of California. In H. V. Daly , J. A. Powell , J. N. Belkin , R. M. Bohart , D. P. Furman , J. D. Pinto , & R. W. Thorp (Eds.), Bulletin of the California insect survey (Vol. 23). University of California Press.

[ece39778-bib-0096] Urban, M. C. (2018). Escalator to extinction. Proceedings of the National Academy of Sciences of the United States of America, 115(47), 11871–11873. 10.1073/pnas.1817416115 30397152 PMC6255192

[ece39778-bib-0097] Vasconcellos, M. M. , Colli, G. R. , Weber, J. N. , Ortiz, E. M. , Rodrigues, M. T. , & Cannatella, D. C. (2019). Isolation by instability: Historical climate change shapes population structure and genomic divergence of treefrogs in the neotropical Cerrado savanna. Molecular Ecology, 28(7), 1748–1764. 10.1111/mec.15045 30742734

[ece39778-bib-0098] Vignali, S. , Barras, A. G. , Arlettaz, R. , & Braunisch, V. (2020). SDMtune: An R package to tune and evaluate species distribution models. Ecology and Evolution, 10(20), 11488–11506. 10.1002/ece3.6786 33144979 PMC7593178

[ece39778-bib-0099] Wickham, H. (2016). ggplot2: Elegant graphics for data analysis. Springer‐Verlag. Retrieved from. https://ggplot2.tidyverse.org

[ece39778-bib-0100] Williams, P. H. (1998). An annotated checklist of bumble bees with an analysis of patterns of description (Hymenoptera: Apidae, Bombini). Bulletin of the Natural History Museum, 67(1), 79–152.

[ece39778-bib-0101] Williams, P. H. , Bystriakova, N. , Huang, J. , Miao, Z. , & An, J. (2015). Bumblebees, climate and glaciers across the Tibetan plateau (Apidae: *Bombus* Latreille). Systematics and Biodiversity, 13(2), 164–181. 10.1080/14772000.2014.982228

[ece39778-bib-0102] Williams, P. H. , Françoso, E. , Martinet, B. , Orr, M. C. , Ren, Z. , Santos Júnior, J. , Thanoosing, C. , & Vandame, R. (2022). When did bumblebees reach South America? Unexpectedly old montane species may be explained by Mexican stopover (Hymenoptera: Apidae). Systematics and Biodiversity, 20(1), 1–24.

[ece39778-bib-0103] Williams, P. H. , Lobo, J. M. , & Meseguer, A. S. (2018). Bumblebees take the high road: Climatically integrative biogeography shows that escape from Tibet, not Tibetan uplift, is associated with divergences of present‐day *Mendacibombus* . Ecography, 41(3), 461–477. 10.1111/ecog.03074

