# Supplementary material for: Whole genome demographic models indicate divergent effective population size histories shape contemporary genetic diversity gradients in a montane bumble bee
Source: Ecol Evol. 2023 Jan 31;13(2):e9778. doi: 10.1002/ece3.9778 (PMC9889631; doi:10.1002/ece3.9778)
Supplement: Supplementary file 1 — Appendix S1 [file ECE3-13-e9778-s001.zip › ECE3_9778_Lozier_VancDemographicModeling_SUPPLEMENTARY DATA_complete_REVISED.docx]

**SUPPLEMENTARY DATA FOR**

**Whole genome demographic models indicate divergent effective population size histories shape contemporary genetic diversity gradients in a montane bumble bee**

Jeffrey D Lozier, Sam D. Heraghty, James P. Strange

Table S1. Sample Site Information for genomes used in this study

| **Site** | **State** | **Region: Population Group** | **No. Genomes** | **Genome Type** | **Latitude** | **Longitude** | **Elev (m)** |
| --- | --- | --- | --- | --- | --- | --- | --- |
| CA17.2015 | CA | Southern: Southern CA | 3 | Moderate Coverage | 37.217 | -119.196 | 2223 |
| CA16.2015 | CA | Southern: Southern CA | 2 | Moderate Coverage | 37.288 | -119.103 | 2747 |
| CA24.2015 | CA | Southern: Southern CA | 4 | Moderate Coverage | 38.333 | -119.643 | 2902 |
| CA25.2015 | CA | Southern: Southern CA | 4 | Moderate Coverage | 39.230 | -120.142 | 2361 |
| CA02.2014 | CA | Mid-range: Northern CA | 6 | Moderate Coverage | 41.362 | -122.201 | 2377 |
| OR11.2014 | OR | Mid-range: Southern OR | 3 | Moderate Coverage | 42.067 | -122.683 | 1754 |
| OR09.2015 | OR | Northern: Northern OR | 1 | Moderate Coverage | 45.256 | -121.712 | 1047 |
| OR03.2016 | OR | Northern: Northern OR | 3 | Moderate Coverage | 45.334 | -121.708 | 1835 |
| WA10.2016 | WA | Northern: WA | 1 | Moderate Coverage | 47.535 | -120.678 | 1228 |
| WA15.2016 | WA | Northern: WA | 1 | Moderate Coverage | 47.801 | -121.058 | 1390 |
| WA16.2016 | WA | Northern: WA | 3 | Moderate Coverage | 47.804 | -121.068 | 1299 |
| WA17.2016 | WA | Northern: WA | 3 | Moderate Coverage | 47.809 | -120.714 | 584 |
| WA12.2016 | WA | Northern: WA | 4 | Moderate Coverage | 48.282 | -117.582 | 1549 |
| WA11.2016 | WA | Northern: WA | 2 | Moderate Coverage | 48.299 | -117.413 | 840 |
| WA14.2016 | WA | Northern: WA | 2 | Moderate Coverage | 48.642 | -120.404 | 1892 |
| CA14.2015 | CA | Southern: Southern CA | 1 | High Coverage Genome | 36.597 | -118.736 | 2214 |
| OR06.2016 | OR | Northern: Northern OR | 1 | High Coverage Genome | 45.256 | -121.712 | 1047 |
| JS.SJ01.2018 | WA | San Juan Island | 1 | High Coverage Genome | 48.535 | -123.044 | 49 |

Table S2. Sample Information for genomes used in this study, with groupings into regions used used in the study, coverage group information (see Figure 1B, C), mean sequencing depth from bam files used for PSMC and MSMC (Samtools -depth function), and Accession information for the National Center for Biotechnology Information (NCBI) Sequence Read Archive.

| **Sample** | **Site** | **Region Population Group** | **Coverage Category** | **Mean Seq Depth** | **NCBI Accession** |
| --- | --- | --- | --- | --- | --- |
| JDL1245 | CA14.2015 | Southern: Southern CA | High | 81.94 | SRX7986195 |
| JDL3160 | OR06.2016 | Northern: Northern OR | High | 76.71 | SAMN29751581 |
| JDL3191 | JS.SJ01.2018 | San Juan Island | High | 79.66 | SRX7270433 |
| JDL1303 | CA17.2015 | Southern: Southern CA | Moderate | 19.31 | SAMN29751564 |
| JDL1305 | CA17.2015 | Southern: Southern CA | Moderate | 21.97 | SAMN29751483 |
| JDL1308 | CA17.2015 | Southern: Southern CA | Moderate | 22 | SAMN29751504 |
| JDL1293 | CA16.2015 | Southern: Southern CA | Moderate | 20.76 | SAMN29751511 |
| JDL1294 | CA16.2015 | Southern: Southern CA | Moderate | 18.42 | SAMN29751541 |
| JDL1388 | CA24.2015 | Southern: Southern CA | Moderate | 18 | SAMN29751498 |
| JDL1389 | CA24.2015 | Southern: Southern CA | Moderate | 18.56 | SAMN29751546 |
| JDL1396 | CA24.2015 | Southern: Southern CA | Moderate | 18.05 | SAMN29751501 |
| JDL1400 | CA24.2015 | Southern: Southern CA | Moderate | 21.97 | SAMN29751527 |
| JDL1406 | CA25.2015 | Southern: Southern CA | Moderate | 18.09 | SAMN29751510 |
| JDL1417 | CA25.2015 | Southern: Southern CA | Moderate | 23.78 | SAMN29751503 |
| JDL1418 | CA25.2015 | Southern: Southern CA | Moderate | 19.94 | SAMN29751461 |
| JDL1419 | CA25.2015 | Southern: Southern CA | Moderate | 28.45 | SAMN29751492 |
| JDL695 | CA02.2014 | Mid-range: Northern CA | Moderate | 25.6 | SAMN29751522 |
| JDL696 | CA02.2014 | Mid-range: Northern CA | Moderate | 20.77 | SAMN29751550 |
| JDL697 | CA02.2014 | Mid-range: Northern CA | Moderate | 27.94 | SAMN29751496 |
| JDL698 | CA02.2014 | Mid-range: Northern CA | Moderate | 20.49 | SAMN29751573 |
| JDL700 | CA02.2014 | Mid-range: Northern CA | Moderate | 21.13 | SAMN29751547 |
| JDL702 | CA02.2014 | Mid-range: Northern CA | Moderate | 29.49 | SAMN29751484 |
| JDL913 | OR11.2014 | Mid-range: Southern OR | Moderate | 22.53 | SAMN29751464 |
| JDL915 | OR11.2014 | Mid-range: Southern OR | Moderate | 25.07 | SAMN29751514 |
| JDL923 | OR11.2014 | Mid-range: Southern OR | Moderate | 25.49 | SAMN29751566 |
| JDL1539 | OR09.2015 | Northern: Northern OR | Moderate | 19.56 | SAMN29751555 |
| JDL3116 | OR03.2016 | Northern: Northern OR | Moderate | 26.44 | SAMN29751576 |
| JDL3117 | OR03.2016 | Northern: Northern OR | Moderate | 18.64 | SAMN29751544 |
| JDL3121 | OR03.2016 | Northern: Northern OR | Moderate | 21.78 | SAMN29751532 |
| JDL2921 | WA10.2016 | Northern: WA | Moderate | 20.45 | SAMN29751476 |
| JDL3049 | WA15.2016 | Northern: WA | Moderate | 22.44 | SAMN29751466 |
| JDL3060 | WA16.2016 | Northern: WA | Moderate | 22.98 | SAMN29751519 |
| JDL3071 | WA16.2016 | Northern: WA | Moderate | 19.82 | SAMN29751557 |
| JDL3072 | WA16.2016 | Northern: WA | Moderate | 20.42 | SAMN29751507 |
| JDL3077 | WA17.2016 | Northern: WA | Moderate | 25.94 | SAMN29751554 |
| JDL3078 | WA17.2016 | Northern: WA | Moderate | 19.45 | SAMN29751488 |
| JDL3085 | WA17.2016 | Northern: WA | Moderate | 20.62 | SAMN29751536 |
| JDL3018 | WA12.2016 | Northern: WA | Moderate | 32.7 | SAMN29751469 |
| JDL3022 | WA12.2016 | Northern: WA | Moderate | 22.35 | SAMN29751490 |
| JDL3030 | WA12.2016 | Northern: WA | Moderate | 22.5 | SAMN29751568 |
| JDL3031 | WA12.2016 | Northern: WA | Moderate | 22.34 | SAMN29751520 |
| JDL3001 | WA11.2016 | Northern: WA | Moderate | 24.54 | SAMN29751460 |
| JDL3006 | WA11.2016 | Northern: WA | Moderate | 25.82 | SAMN29751559 |
| JDL3035 | WA14.2016 | Northern: WA | Moderate | 27.15 | SAMN29751470 |
| JDL3046 | WA14.2016 | Northern: WA | Moderate | 22.47 | SAMN29751558 |

|  | **Heterozygosity** | | | | **PSMC Bottleneck Size** | | | | **PSMC Bottleneck Size** | | | | **PSMC Bottleneck Size** | | | |
| --- | --- | --- | --- | --- | --- | --- | --- | --- | --- | --- | --- | --- | --- | --- | --- | --- |
| *Predictors* | *Est* | *SE* | *t* | *p* | *Est* | *SE* | *t* | *p* | *Est* | *SE* | *t* | *p* | *Est* | *SE* | *t* | *P* |
| (Intercept) | 0.002 | 0.000 | 21.458 | **<0.001** | 18.975 | 6.248 | 3.037 | **0.009** | 5.428 | 3.248 | 1.671 | 0.121 | 15.289 | 4.348 | 3.516 | **0.004** |
| a) Suitability Difference (current – LGM) | 0.000 | 0.000 | 1.396 | 0.186 | 16.247 | 8.070 | 2.013 | 0.065 |  |  |  |  |  |  |  |  |
| b) Distance to nearest LGM cell with suitability ≥ mean of sample sites (=0.882) [log10] |  |  |  |  |  |  |  |  | 11.468 | 1.421 | 8.069 | **<0.001** |  |  |  |  |
| c) Distance to nearest LGM cell with suitability ≥ mean of 453 niche model presence sites (=0.609) [log10] |  |  |  |  |  |  |  |  |  |  |  |  | 8.969 | 2.351 | 3.816 | **0.002** |
| **Random Effects** | | | | | | | | | | | | | | | | |
| σ^2^ | 0.00 | | | | 6.92 | | | | 6.94 | | | | 6.97 | | | |
| τ_00_ | 0.00 _Site_ | | | | 48.33 _Site_ | | | | 8.47 _Site_ | | | | 28.52 _Site_ | | | |
| ICC | 0.96 | | | | 0.87 | | | | 0.55 | | | | 0.80 | | | |
| N | 15 _Site_ | | | | 15 _Site_ | | | | 15 _Site_ | | | | 15 _Site_ | | | |
| Observations | 42 | | | | 42 | | | | 42 | | | | 42 | | | |
| Marginal R^2^ / Conditional R^2^ | 0.117 /  0.964 | | | | 0.208 /  0.901 | | | | 0.769 /  0.896 | | | | 0.463 /  0.894 | | | |

Table S3: Linear mixed effects models testing the effects on heterozygosity or PCMC-inferred bottleneck size (see Figure S1) from niche stability between current and LGM climate periods (a) and spatial distance from each sampling locality and the closest LGM raster cell with a predicted suitability either equal to the mean of sampled genomes (b) or the mean of all 453 occurrence records used for Maxent modeling (c) in the contemporary time period.

Figure S1. Schematic illustrating the PSMC-inferred glacial bottleneck size (Figure 3, Table S3) from a representative PCMC plot, essentially estimated from curves in Figure 1B as the maximum *N*_e_ at the last interglacial (LIG) and the minimum *N*_e_ at or near the last glacial maximum (LGM). Also shown is a schematic for estimating the climatic suitability difference at each sample site as well as the “minimum distance to a suitable LGM point” metric which is determined as the distance of each point to LGM SDM raster cells with an average suitability of that observed at sampled localities (or natural history occurrence points) in the contemporary climate (Current SDM). See Table S3 for model results.

Figure S2. PSMC curves estimated using a mappability masked genome, indicating little impact on inferred PSMC *N*e (x 10^4^) trajectories (Figure 1B-C).

Figure S3. *N*_e_ trajectories estimated by MSMC2 for populations (some samples from adjacent or equivalent sites sampled in different years pooled, see Table S1), color coded as in Figure 1-3.

Figure S4. Downsampled (N=9 diploid samples per region) ANGSD-estimated Site Frequency Spectra

Figure S5. Ecological Niche Model Summaries from Maxent (maxnet) model outputs, including the distribution of Test (blue points) and Training (red points), ROCs and AUCs for test and training data as well as predictor variable permutation importance and jackknife importance for test and training data.
